# Contrasting ability to take up leucine and thymidine among freshwater bacterial groups: implications for bacterial production measurements

**DOI:** 10.1111/j.1462-2920.2009.02043.x

**Published:** 2010-01

**Authors:** María Teresa Pérez, Paul Hörtnagl, Ruben Sommaruga

**Affiliations:** Laboratory of Aquatic Photobiology and Plankton Ecology, Institute of Ecology, University of InnsbruckTechnikerstrasse 25, 6020 Innsbruck, Austria

## Abstract

We examined the ability of different freshwater bacterial groups to take up leucine and thymidine in two lakes. Utilization of both substrates by freshwater bacteria was examined at the community level by looking at bulk incorporation rates and at the single-cell level by combining fluorescent *in situ* hybridization and signal amplification by catalysed reporter deposition with microautoradiography. Our results showed that leucine was taken up by 70–80% of *Bacteria*-positive cells, whereas only 15–43% of *Bacteria*-positive cells were able to take up thymidine. When a saturating substrate concentration in combination with a short incubation was used, 80–90% of *Betaproteobacteria* and 67–79% of *Actinobacteria* were positive for leucine uptake, whereas thymidine was taken up by < 10% of *Betaproteobacteria* and by < 1% of the R-BT subgroup that dominated this bacterial group. Bacterial abundance was a good predictor of the relative contribution of bacterial groups to leucine uptake, whereas when thymidine was used *Actinobacteria* represented the large majority (> 80%) of the cells taking up this substrate. Increasing the substrate concentration to 100 nM did not affect the percentage of R-BT cells taking up leucine (> 90% even at low concentrations), but moderately increased the fraction of thymidine-positive R-BT cells to a maximum of 35% of the hybridized cells. Our results show that even at very high concentrations, thymidine is not taken up by all, otherwise active, bacterial cells.

## Introduction

The accurate estimation of bacterial growth and production rates is essential for understanding their role in aquatic ecosystems. The two most common methods used to estimate bacterial secondary production measure either the incorporation of labelled thymidine (TdR) into the deoxyribonucleic acid (DNA) or of labelled leucine (Leu) into proteins. An intrinsic assumption in both methods is that all heterotrophic bacteria possess the ability to incorporate the above-mentioned substrates. Some early studies have pointed out that some marine bacteria lack the appropriate enzymes or transport systems to incorporate TdR (Pollard and Moriarty, 1984; Davis, 1989; Jeffrey and Paul, 1990). However since the reference work of Fuhrman and Azam (1982), where all active bacteria in Southern California Bight were found to incorporate TdR, this method became one of the routine procedures to measure bacterial production in aquatic systems. Later studies in the eighties (Novitsky, 1983; Douglas *et al*., 1987) found, on the contrary, higher proportions of bacteria positive for amino acid (glutamate) uptake than for TdR, suggesting that some marine bacterial subpopulations might not incorporate TdR. Pedrós-Alió and Newell (1989) concluded that the assumption of all active bacteria taking up TdR had to be questioned, if not rejected. Despite the numerous studies showing that only a fraction of the bacterial community was actually taking up TdR, there was no possibility at that time to examine whether this fraction remained constant among different phylogenetic groups or whether some bacterial taxa could not incorporate TdR at all.

The development in recent years of protocols combining *in situ* phylogenetic identification (Amann *et al*., 1995; Pernthaler *et al*., 2002) with methods assessing single-cell activity (Lee *et al*., 1999; Ouverney and Fuhrman, 1999; Cottrell and Kirchman, 2000; Teira *et al*., 2004) offers us an excellent tool to re-examine this question.

There is now growing evidence indicating that different phylogenetic groups differ in their ability to utilize specific dissolved organic compounds (Cottrell and Kirchman, 2000; Elifantz *et al*., 2005; Malmstrom *et al*., 2005) or exhibit distinct affinities for a given substrate (Alonso and Pernthaler, 2006). Furthermore, differences in substrate utilization have been found even among distinct lineages within a phylogenetic group (Salcher *et al*., 2008). Thus, it seems even more important to re-assess whether the ability to incorporate the routinely used substrates TdR and Leu is widespread among different bacterial groups. Cottrell and Kirchman (2003) examined this question in the Delaware estuary and found, contrary to their initial hypothesis, that all major bacterial groups were able to take up Leu and TdR. Beside the fact that this study included a low-salinity station, information about TdR uptake by specific freshwater bacterial groups is scarce.

Differences between the marine and freshwater bacterial communities are noteworthy. The former is dominated by *Alphaproteobacteria*, whereas *Betaproteobacteria* and *Actinobacteria* are widespread among the heterotrophic bacterial assemblage from freshwater ecosystems. By contrast, *Cytophaga-*like bacteria are found in both freshwater and marine bacterial communities (Kirchman, 2002).

Here, we examined the uptake of Leu and TdR by freshwater bacterial groups in two alpine lakes, Gossenköllesee (GKS) and Schwarzsee ob Sölden (SOS). Gossenköllesee was sampled in September 2006 at two different depths (1 m and 8.5 m). Schwarzsee ob Sölden was sampled twice at 1 m depth, in September 2006 and in August 2007. In addition to substrate bulk incorporation rates, we examined the ability of specific phylogenetic groups to take up the two substrates by combining fluorescent *in situ* hybridization and signal amplification by catalysed reporter deposition (CARD-FISH) with microautoradiography (MAR). Our results showed that some abundant bacterial groups exhibited a contrasting ability to take up Leu and TdR.

## Results

### Impact of the substrate specific activity on the relative abundance of MAR-positive cells

We tested the influence of the substrate-specific activity on the relative abundance of MAR-labelled cells using TdR with two different specific activities. The percentages of MAR-labelled cells obtained with the low and high specific activity TdR were 19.0% (SD 2.0) and 19.5% (SD 2.1) respectively. By contrast, the incubation with Leu yielded double as much labelled cells (42.6%; SD 1.6). The anova performed detected significant differences among substrates (*P* < 0.001). Both, the low and high specific activity TdR yielded a significantly different per cent of cells labelled compared with the incubation with Leu, but the percentages of cells labelled with either TdR were not significantly different from each other (*t* = 0.379; *P* = 0.715).

### Bacterial community composition

The number of cells detected with the *Bacteria* probe (EUB I-III) was high in both lakes and ranged between 68% and ∼90% of DAPI counts depending on the sample considered (Table 1). *Betaproteobacteria* and *Actinobacteria* dominated the bacterial community of both lakes. In GKS, these two groups together represented ∼40% of DAPI counts and ∼60% of *Bacteria* cells, regardless of the depth considered. *Alphaproteobacteria* and *Cytophaga-*like bacteria were also present, but their contribution to bacterial abundance was more variable. Whereas *Alphaproteobacteria* accounted for ∼10% of DAPI counts at 1 m depth, their contribution at 8.5 m was low (Table 1). At this depth, *Cytophaga*-like bacteria represented a substantial fraction of DAPI counts (17.7%). In SOS, *Betaproteobacteria* and *Actinobacteria* together accounted for > 65% and > 70% of DAPI and *Bacteria* counts respectively. In this lake, *Alphaproteobacteria* was the third most abundant group and accounted for ∼5% of DAPI counts. The relative abundance of *Cytophaga*-like cells was very low in SOS (< 1% DAPI counts). In GKS, > 60% of *Betaproteobacteria* were detected with probe R-BT065, this percentage was even higher in SOS (September 2006 and August 2007) where > 80% of *Betaproteobacteria* cells belonged to the R-BT subgroup.

**Table 1:** Bacterial abundance (BA), temperature (T), bulk incorporation rates for leucine (BP Leu) and thymidine (BP TdR) and the relative abundance of the main bacterial groups expressed as percentage of DAPI counts.

Sample	T (°C)	BA 10^5^ cells ml^−1^	BP Leu pmol l^−1^ h^−1^	BP TdR pmol l^−1^ h^−1^	EUB %	ALFA %	BET %	RBT %	CF %	HGC %
GKS 1	11.2	4.80 ± 0.53	163 ± 9.43	4.56 ± 0.94	68.0 ± 3.21	9.72 ± 1.64	18.1 ± 1.83	12.0 ± 1.48	8.11 ± 1.35	20.0 ± 4.56
GKS 8.5	8.7	5.40 ± 0.27	164 ± 15.7	3.59 ± 0.78	71.5 ± 2.44	3.61 ± 0.58	20.8 ± 2.20	12.7 ± 1.02	17.7 ± 1.74	19.9 ± 3.76
SOS 06	7.5	4.80 ± 0.59	61.3 ± 2.85	2.48 ± 0.24	82.9 ± 7.39	5.13 ± 0.59	29.0 ± 3.12	23.9 ± 3.21	0.76 ± 0.18	37.7 ± 4.22
SOS 07	9.2	6.24 ± 0.38	27.9 ± 2.53	1.73 ± 0.26	89.7 ± 3.51	4.90 ± 0.65	41.7 ± 3.72	33.2 ± 6.65	0.49 ± 0.12	23.3 ± 3.46

EUB (*Bacteria*), ALFA (*Alphaproteobacteria*), BET (*Betaproteobacteria*), RBT (R-BT subgroup of *Betaproteobacteri*a), CF (*Cytophaga*-like bacteria), HGC (*Actinobacteria*). GKS 1 and GKS 8.5 are samples collected in September 2006 in GKS at 1 and 8.5 m depth respectively. SOS 06 and SOS 07 are samples collected in SOS at 1 m depth in September 2006 and August 2007 respectively.

### Bacterial bulk incorporation rates

Bulk incorporation rates for Leu and TdR were higher in GKS than in SOS (Table 1). In GKS, Leu incorporation rates were almost identical, regardless of the depth considered, whereas TdR incorporation was slightly lower at 8.5 m depth. Leu incorporation rates in SOS ranged from 27.9 to 61.3 pmol l^−1^ h^−1^ depending on the sample, whereas TdR incorporation rates were less variable ranging from 1.73 to 2.46 pmol l^−1^ h^−1^. The Leu/TdR ratio ranged from 46 in GKS at 8.5 m depth to 16 in August 2007 in SOS (1 m depth).

### Substrate uptake by individual bacterial groups

**Fig. 1:**
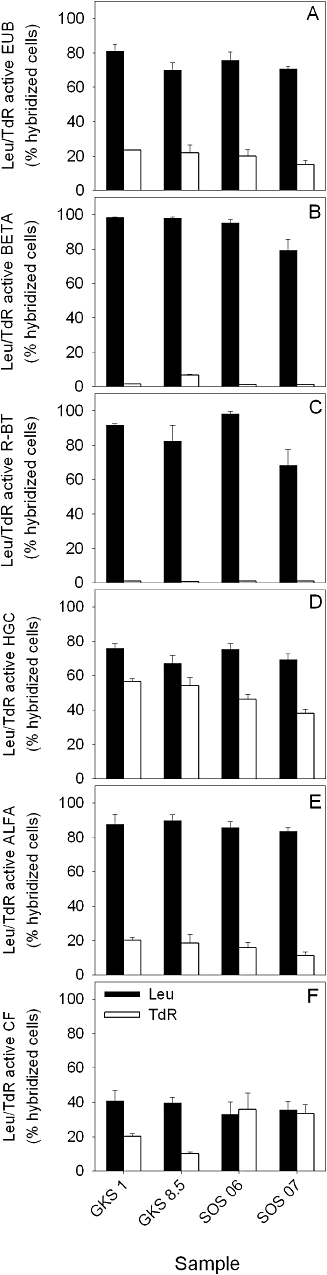
Percentage of cells active for Leu (solid bars) and TdR (open bars) uptake within the most abundant bacterial groups: EUB (*Bacteria*; A), BETA (*Betaproteobacteria*; B), R-BT (subgroup of *Betaproteobacteria*; C), HGC (*Actinobacteria*; D), ALFA (*Alphaproteobacteria*; E) and CF (*Cytophaga*-like cells; F). GKS 1 and GKS 8.5 represent samples collected in September 2006 in GKS at 1 and 8.5 m depth respectively. SOS 06 and SOS 07 represent samples collected in SOS at 1 m depth in September 2006 and August 2007 respectively. Values are mean of three replicates ± 1 SD.

Between 70% and 80% of cells hybridized with the *Bacteria* probe were able to take up Leu, whereas only 15–24% of *Bacteria* took up TdR in the lakes studied (Fig. 1A). Eighty to ninety-eight per cent of *Betaproteobacteria* cells took up Leu. By contrast, only 1–7% of *Betaproteobacteria* showed TdR uptake (Fig. 1B). Within the R-BT subgroup to which belonged most of the *Betaproteobacteria* in both lakes, 70–98% of the hybridized cells took up Leu, but < 1% of R-BT were able to take up TdR. Between 67% and 76% of cells belonging to the class *Actinobacteria* took up Leu and 38–57% were positive for TdR uptake (Fig. 1D). On average, 85% of the cells hybridizing with the probe targeting *Alphaproteobacteria* were active for Leu uptake and between 11% and 20% for TdR uptake. In GKS, *Cytophaga*-like bacteria differed in their ability to take up Leu and TdR, whereas 40% of the hybridized cells incorporated Leu, only 10–20% did incorporate TdR (Fig. 1F). In SOS, ∼34% of *Cytophaga*-like cells took up either TdR or Leu.

### Contribution of the different bacterial groups to Leu and TdR uptake

**Fig. 2:**
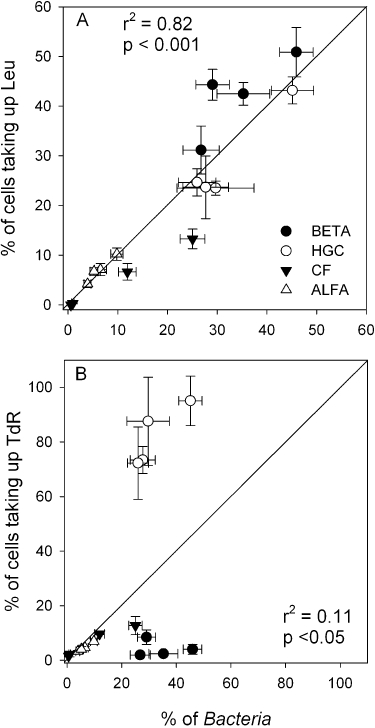
Relative contribution of *Betaproteobacteria* (BETA), *Actinobacteria* (HGC), *Cytophaga*-like bacteria (CF) and *Alphaproteobacteria* (ALFA) to Leu (A) or TdR (B) uptake plotted against their relative abundance among *Bacteria*. Values are mean of three replicates ± 1 SD. The diagonal line represents a 1:1 relationship.

*Betaproteobacteria* made the highest contribution to Leu uptake. Their members represented 30–50% of the cells taking up Leu (Fig. 2A). The second group in importance for Leu uptake was *Actinobacteria,* representing between 20% and 40% of all cells taking up Leu, depending on the sample considered. The contribution of *Cytophaga*-like bacteria to Leu uptake was only relevant in GKS, where they represented 6–13% of cells taking up Leu depending on the depth considered. *Alphaproteobacteria* represented between 4% and 10% of cells positive for Leu uptake.

When looking at TdR uptake, it was striking that in all samples *Actinobacteria* represented the large majority (72–95%) of TdR-positive cells (Fig. 2B). The contribution of the other bacterial groups considered in this study was modest and generally accounted for less than 10% of cells taking TdR.

### Effect of the substrate concentration on the Leu and TdR uptake by *Betaproteobacteria*

**Fig. 3:**
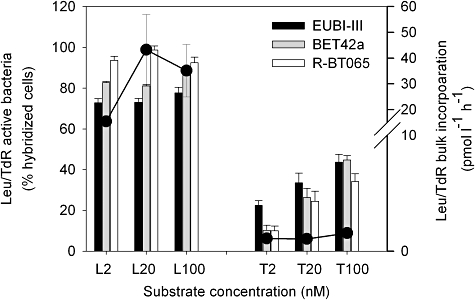
Fractions of *Bacteria* (probe EUB I-III), *Betaproteobacteria* (probe BET42a) and R-BT cells (probe R-BT065) positive for Leu (left) and TdR (right) uptake at different substrate concentrations. Dots and lines show the corresponding bulk incorporation rates of Leu and TdR by the whole bacterial assemblage.

We included an additional experiment in order to examine whether the contrasting ability of *Bacteria* in general and more specifically of *Betaproteobacteria* to take up Leu and TdR was concentration-dependent. Figure 3 summarizes the outcome of this experiment. Leucine bulk incorporation rates increased significantly at concentrations above 2 nM. Nevertheless, the three different concentrations (i.e. 2, 20, 100 nM) tested did not yield significantly different fractions of *Betaproteobacteria* with visible Leu uptake. Regardless of the Leu concentration, we detected > 92% of R-BT cells taking up Leu.

Increasing TdR concentrations did not translate into significant differences in bulk incorporation rates by the whole bacterial assemblage (Fig. 3), but yielded increasing fractions of *Bacteria* and *Betaproteobacteria* positive for TdR uptake. Even when TdR was added at a 100 nM concentration, only 44% of *Bacteria* and *Betaproteobacteria* and 35% of R-BT cells took up TdR. By contrast at that concentration, 86% of *Betaproteobacteria* and 93% of the R-BT cells were positive for Leu uptake.

## Discussion

Our results clearly show that members of the main bacterial groups present in these two lakes differ in their ability to take up the two substrates commonly used to assess bacterial production in aquatic systems.

### Influence of substrate-specific activity on the percentage MAR-positive cells

Earlier studies involving MAR claimed that it is inappropriate to compare the percentage of MAR-labelled cells between two samples, when one sample contains more radioactivity per cell (on average) than the other (Fuhrman and Azam, 1982). A homogeneous distribution of radioactivity per cell among samples is very difficult to achieve in practice, particularly when working with different substrates. The amount of radioactivity incorporated per cell within a sample is not only related to the specific activity of the substrate, but largely depends on the uptake kinetics of the bacterial assemblage for a given substrate. Our incubations with TdR, with either a low or a high specific activity, yielded a statistically indistinguishable number of cells labelled, despite the fact that the ‘cold’ sample contained only 30% of the radioactivity of the sample incubated with high specific activity TdR. This point illustrates that comparison among samples with different radioactivity levels is possible, if the exposure time is adjusted to maximize the detection of labelled cells.

### Substrate uptake by individual bacterial groups

A contrasting ability to take up Leu and TdR was found not only for the bacterial assemblage as a whole, but almost within every bacterial group we considered. In the lakes we sampled, *Betaproteobacteria* co-dominated the bacterial community together with *Actinobacteria*. Both groups together comprised more than half of the cells detected with the probe EUB I-III targeting *Bacteria*. When a single substrate concentration (20 nM) in combination with a short incubation time was used, the majority of *Betaproteobacteria* was able to take up Leu (on average 92% of hybridized cells), but they showed a limited capability to take up TdR (on average < 3% of hybridized cells). Previous studies in estuarine waters reported 32% of *Betaproteobacteria* cells taking up Leu at the low salinity station of the Pearl River estuary (Zahng *et al*., 2006) and between 23% and 33% of *Betaproteobacteria* were positive for Leu uptake in Delaware River (Cottrell and Kirchman, 2004). Unlike our results, the fraction of *Betaproteobacteria* positive for TdR uptake in the latter river, though variable, was sometimes higher than that positive for Leu uptake (Cottrell and Kirchman, 2003; 2004).

The importance of *Betaproteobacteria* in freshwater systems is well documented since *in situ* quantitative methods have been employed (Alfreider *et al*., 1996; Šimek *et al*., 1997; Methé*et al*., 1998). Nevertheless, this subclass of *Proteobacteria* is composed of several lineages that might differ in their capacity to incorporate different substrates. In the meso-oligotrophic Piburger See (Austria), Salcher and colleagues (2008) reported that members of three different *Betaproteobacteria* lineages differed in their ability to take up an amino acid mixture. These authors found that, 80% of Beta I cells (detected with probe R-BT065), 36% of Beta II cells and 13% of Beta IV cells were positive for amino acid uptake. The contrasting behaviour of these bacterial lineages to take up an identical substrate could explain the discrepancy between our results and those of Cottrell and Kirchman (2003; 2004), if other *Betaproteobacteria* lineages in the Delaware River were relatively more abundant than the R-BT subgroup that comprised the majority of *Betaproteobacteria* in our samples (> 60% in GKS and > 80% in SOS). In agreement with the results of Salcher and colleagues (2008), we found that on average 85% of R-BT cells took up Leu. However, this *Betaproteobacteria* subgroup showed a limited capacity to take up TdR as shown by the fact that the percentage of TdR-labelled cells within this subgroup was always much lower than the percentage of Leu-labelled cells.

Among the other bacterial groups we examined the fraction of hybridized cells taking up TdR was generally smaller than that of cells taking up Leu, except for the *Cytophaga*-like cells in SOS. The proportion of cells taking up Leu within this group was about half of the other bacterial groups, which is in agreement with findings indicating that members of the *Cytophaga*-like bacteria are not specialized in consuming low-molecular-weight dissolved organic matter (Cottrell and Kirchman, 2000; Kirchman, 2002). However, it is noteworthy that a substantial part of the *Cytophaga*-like cells took up TdR in SOS. The smallest differences in the proportions of Leu- and TdR-positive cells were found within the class *Actinobacteria*. In our samples 38–57% of *Actinobacteria* took up TdR, which was well above the average of TdR-active *Bacteria*. In the Delaware River, Kirchman and colleagues (2005) also found the fraction of TdR active *Actinobacteria* to be higher than the corresponding fraction of active prokaryotes.

### Effect of substrate concentration on Leu and TdR uptake by *Betaproteobacteria*

Given the ubiquity and importance of *Betaproteobacteria* in freshwater systems, we further tested whether their disproportional response to the two substrates was concentration-dependent. In marine waters, it has been shown that the relative abundance of Leu-active cells increased with increasing substrate concentration for some particular bacterial clades (Alonso and Pernthaler, 2006).

In this study we found that the fraction of Leu-active *Betaproteobacteria* and especially of R-BT cells was always larger than the fraction of active *Bacteria* and did not change with the Leu concentration used, pointing out that this group plays a very important role in Leu uptake and potentially in Leu assimilation over a wide range of concentrations. A similar finding was observed in marine waters for the *Roseobacter* clade (Alonso and Pernthaler, 2006). However, the fraction of TdR active *Betaproteobacteria* and particularly of its R-BT subgroup increased with the TdR concentration offered, but it was always much smaller than the corresponding fraction of Leu active cells. Even after a long incubation and a high TdR concentration (100 nM) only 35% of R-BT cells took up TdR, whereas under similar conditions 93% of cells showed Leu uptake. This is striking considering that in the studied lakes a TdR concentration of 2 nM is saturating.

Yet, the question is why such a high proportion of the otherwise very active *Betaproteobacteria* and particularly R-BT cells in our study were unable to incorporate TdR. One possibility is that this subgroup was out of balanced growth conditions (Chin-Leo and Kirchman, 1990); that is, cells were growing (incorporating leucine) but not synthesizing DNA. However, the R-BT subgroup are fast-growing bacteria (Šimek *et al*., 2006) exhibiting doubling times as short as 8 h (Šimek *et al*., 2005) and are able to rapidly overgrow other bacterial groups under experimental and natural conditions (Šimek *et al*., 2005; Pérez and Sommaruga, 2006; Salcher *et al*., 2007). Thus, it seems unlikely that unbalanced growth of the R-BT subgroup is responsible for the observed pattern, particularly because this state is necessarily transient.

Another possibility is that certain subpopulations within this subgroup lacked the appropriate enzymes to transport this substrate or that they preferred to use the *de novo* pathway as argued by Pedrós-Alió and Newell (1989) to explain why active bacteria in their samples did not take up TdR. The incapacity of some bacterial isolates to incorporate TdR has already been reported. For example, representatives of *Pseudomonas* lack thymidine kinase (reviewed in Robarts and Zohary, 1993), whereas some *Vibrio* do not have TdR transport systems (Jeffrey and Paul, 1990). Usually, if enough exogenous TdR is added to the samples the *de novo* pathway is assumed to be blocked. Nevertheless, it is known that increasing the concentration of external TdR also inhibits a step of its own conversion into thymidine triphosphate, and therefore, it is recommended to use the lowest possible concentration of TdR (O'Donovan, 1978). However, this concentration might not totally inhibit the *de novo* pathway. The increasing fraction of R-BT cells taking up TdR at increasing substrate concentrations might be considered as evidence in favour of this hypothesis. Further, as the R-BT subgroup of *Betaproteobacteria* is not a monospecific cluster and includes a relatively large set of sequences (Šimek *et al*., 2001), it is likely that different R-BT subpopulations in our samples behaved differently. For instance, some subpopulations could have been taking up TdR at any concentration offered whereas others did not take it up at all, and in other subpopulations the salvage pathway might have been induced by the very high TdR concentrations offered.

### Contribution to Leu and TdR uptake

The contrasting ability among bacterial groups to take up Leu and TdR translated into a different contribution of those groups to the total community of substrate-active cells. On average, *Actinobacteria* were responsible for 82% of TdR uptake. Only few studies have examined the contribution of this bacterial group to the uptake of TdR. In the study of Kirchman and colleagues (2005), 48–65% of TdR uptake was due to *Actinobacteria*. This points out to a major role of this bacterial group in TdR incorporation. Similarly, in the study of Warnecke and colleagues (2005) using the halogenated thymidine analogue 5-Bromo-2′deoxyuridine (BrdU), the relative abundance of BrdU-positive *Actinobacteria* usually matched that of BrdU-positive *Bacteria*, except in three lakes, where the proportion of BrdU-positive *Actinobacteria* exceeded the one of BrdU-positive *Bacteria*. In these lakes, the contribution of *Actinobacteria* to DNA synthesis rates was higher than expected by their relative abundance within the bacterial assemblage, which was also the case in our study (Fig. 2B). However, it should be noted that uridine and thymidine are taken up into the cells by different transport systems.

Cell abundance was a bad predictor of the contribution of different bacterial groups to TdR uptake for the two most abundant phylogenetic groups, whereas the contribution of minor phylogenetic groups to TdR uptake matched the 1:1 relationship (Fig. 2B). Bacterial abundance was a better predictor of the contribution of a particular group to Leu uptake than it was in the case of TdR, although it slightly underestimated the contribution of *Betaproteobacteria* in some cases and overestimated that of *Cytophaga*-like cells in others (Fig. 2A). Although the relationship was significant (*P* < 0.05), bacterial community composition explained only 11% of the variability in TdR uptake (Fig. 2B), which was substantially less than in the study of Cottrell and Kirchman (2003). When Leu was used as substrate, 82% of the variability in Leu uptake was explained by the composition of the bacterial assemblage (Fig. 2A). This is in agreement with the results of Zahng and colleagues (2006) in the Pearl River estuary, but in our case, the bacterial community structure explained more of the variability in Leu uptake than in the Delaware River (Cottrell and Kirchman, 2003).

### Implications for bacterial production measurements

Our results showed that some abundant freshwater bacterial groups strongly differ in their ability to take up Leu and TdR in the concentration range usually used for bacterial production measurements**.** Thus, the latter substrate should be employed with caution in ecosystems where *Betaproteobacteria* and particularly its R-BT subgroup are numerically important. At present, there is only limited information on the occurrence and relative abundance of the R-BT subgroup in freshwaters. Nevertheless, the data available suggest that members of this subgroup are widespread as they have been found in a mesoeutrophic drinking reservoir (Šimek *et al*., 2001), in several oligotrophic high-mountain lakes (Pernthaler *et al*., 1998; Warnecke *et al*., 2005; Pérez and Sommaruga, 2006) in a mesotrophic lake (Posch *et al*., 2007; Salcher *et al*., 2008) and recently in a subtropical lagoon (Alonso *et al*., 2009)*.* Our results also suggest that discrepancies between bacterial biomass production measured with Leu and TdR might not only be due to unbalanced growth (Chin-Leo and Kirchman, 1990) or physiological stress (Ducklow, 2000) as it has been often suggested, but might as well reflect differences in bacterial community composition.

## Experimental procedures

### Sampling sites and sample collection

Water samples were collected from the alpine lakes GKS and SOS. These lakes are located in the Austrian Alps above the treeline at 2417 m and 2799 m (above sea level) respectively, As most high-mountain lakes in the region, they have a small catchment area (30 ha GKS and 18 ha SOS), which is mainly composed by bare rocks. Other characteristics of the lakes sampled can be found in Laurion and colleagues (2000) and in Sommaruga and Augustin (2006).

Triplicate water samples were collected in the central part of GKS (max. depth 9.9 m) at 1 m and at 8.5 m depth in September 2006. In SOS triplicate samples were collected twice in September 2006 and in August 2007 at 1 m depth. At every sampling date basic physico-chemical parameters (temperature, pH and dissolved organic carbon concentration) were measured. Samples for bacterial abundance were fixed with formaldehyde (2% final concentration) and the concentration of bacteria estimated by epifluorescence microscopy after DAPI staining. Bulk measurements of substrate incorporation and MAR incubations were run as explained in the following sections.

### Bulk incorporation rates of Leu and TdR

Bacterial bulk production was estimated by measuring incorporation of two different substrates: (i) ^3^H-TdR (specific activity: 27 Ci mmol^−1^; 20 nM final concentration) and (ii) ^3^H-Leu (specific activity 63 Ci mmol^−1^; 20 nM final concentration). Triplicate 10–15 ml samples plus one formaldehyde-killed blank were incubated at *in situ* temperature in the dark for 1 h. Incubations were stopped by adding formaldehyde at 2% final concentration. Subsequently, the samples were filtered through 0.22 μm Millipore GTTP membrane filters and rinsed twice with 10 ml of 5% trichloroacetic acid for 5 min. Filters were dissolved in 6 ml of scintillation cocktail (Ready-safe, Beckman Coulter) and the radioactivity assessed after 15 h.

### MAR

Samples collected in GKS in September 2006 and in SOS in September 2006 and August 2007 were incubated with Leu and TdR (specific activities as in the former section) under the same conditions as for bacterial production in order to assess which bacterial groups contributed to bulk substrate incorporation.

In October 2008, we performed an experiment in order to test whether the specific activity of a given substrate could influence the relative abundance of cells incorporating that substrate. For that purpose, five replicate water samples collected at the surface of GKS (0.5 m) were incubated with labelled TdR having either a low (27 Ci mmol^−1^) or a high specific activity (86 Ci mmol^−1^), as well as with Leu (63 Ci mmol^−1^). In all three cases the final concentration of the substrate was 20 nM. The incubations were run for 1 h in the dark at *in situ* temperature.

In March 2009, an additional experiment was run to test for concentration-dependent uptake of both, Leu and TdR by *Betaproteobacteria* and its R-BT subgroup. Water was collected in GKS under the ice cover at 1 m depth. Incubations were run for 5 h at 12°C with Leu (62 Ci mmol^−1^) and TdR (79 Ci mmol^−1^) at three different concentrations (2, 20 and 100 nM). For every concentration triplicate samples were used.

Incubations were stopped by adding formaldehyde (2% final concentration). Samples were kept at 4°C overnight and filtered on the next day onto 0.22 μm polycarbonate white filters (GTTP, Millipore), rinsed with 0.22 μm filtered Milli-Q water, air-dried and then stored at −20°C until further analysis. Samples were subjected to MAR as described by Tabor and Neihof (1982) after CARD-FISH was performed. Exposure times for every substrate, concentration and specific activity were determined empirically in a preliminary experiment. The optimum exposure time was assessed by monitoring the detection of positive cells for a given substrate until a maximum was reached. After exposure, slides were developed according to the manufacturer instructions and mounted with an anti-fading solution containing DAPI (final concentration of 1 μg ml^−1^). Slides were stored frozen until microscopic analysis.

Cells were counted in at least 20 randomly selected microscopic fields. For every field four different counts were recorded: (i) DAPI positive cells, (ii) probe-specific positive cells, (iii) DAPI + MAR positive cells and (iv) probe-specific + MAR positive cells. Routinely at least 350 DAPI-stained cells were counted per sample or 1000 DAPI-stained cells if the calculated relative abundance was < 1%.

### CARD-FISH

CARD-FISH was performed according to Pernthaler and colleagues (2002) using the modified permeabilization protocol of Sekar and colleagues (2003) for freshwater bacteria. We used six different horseradish peroxidase-labelled oligonucleotide probes: probe EUB I-III targeting most *Bacteria* (Daims *et al*., 1999), ALF968 (Neef, 1997) for *Alphaproteobacteria*, BET42a (Manz *et al*., 1992) for *Betaproteobacteria*, R-BT065 for the R-BT subgroup of *Betaproteobacteri*a (Šimek *et al*., 2001), HGC69a (Roller *et al*., 1994) for *Actinobacteria* and CF319a (Manz *et al*., 1996) for *Cytophaga*-like bacteria within the *Bacteroidetes*. All hybridizations were conducted during 5 h at 35°C, followed by 30 min amplification. Formamide concentration in the hybridization buffer was always 55% excepting for probe HGC69a which needed 35% formamide concentration.
